# Nanometric resolution magnetic resonance imaging methods for mapping functional activity in neuronal networks

**DOI:** 10.1016/j.mex.2016.04.003

**Published:** 2016-04-16

**Authors:** Albert Boretti, Stefania Castelletto

**Affiliations:** a Department of Mechanical and Aerospace Engineering (MAE), Benjamin M. Statler College of Engineering and Mineral Resources, West Virginia University (WVU), P.O. Box 6106, 325 Engineering Sciences Building, Morgantown, WV 26506, United States; b School of Engineering, RMIT University, Bundoora, VIC 3083, Australia

**Keywords:** Nano Fourier magnetic resonance imaging, Nitrogen vacancy in diamond, Optical detection, Magnetic resonance imaging, Neuronal networks, Nano scale, Super-resolution microscopy

## Abstract

This contribution highlights and compares some recent achievements in the use of k-space and real space imaging (scanning probe and wide-filed microscope techniques), when applied to a luminescent color center in diamond, known as nitrogen vacancy (NV) center. These techniques combined with the optically detected magnetic resonance of NV, provide a unique platform to achieve nanometric magnetic resonance imaging (MRI) resolution of nearby nuclear spins (known as nanoMRI), and nanometric NV real space localization.

• Atomic size optically detectable spin probe.
• High magnetic field sensitivity and nanometric resolution.
• Non-invasive mapping of functional activity in neuronal networks.

## Highlights

• NV in diamond acts as an atomic size optically detectable spin probe.
• The probe combines high magnetic field sensitivity and nanometric resolution.
• Non-invasive mapping of functional activity in neuronal networks is one application.
• Synapse scale resolution ∼10 nm but circuit scale FoV >1 mm is possible.

## Methods details

A nitrogen vacancy (NV) center in diamond [1], [2], [3] acts as an atomic size optically detectable electron spin probe with the ability to sense local small magnetic fields due to other electrons or nuclear spins at nanometric distance. Owed to the NV spin dependent fluorescence, its electronic spin state can be both readout and initialized optically. The application of microwave frequency pulses on its optically initialized state then permits the coherent control of its spin state at the single defect level, when weakly coupled with its surrounding nanometric environment. Thus the NV can be used directly as a sensor. Microwave sequences adapted from nuclear magnetic resonance (NMR) methods allow detecting alteration in NV spin dephasing time originated from a dilute concentration of nuclear spins producing small magnetic field around the probe. Compared to other probes, this defect allows a wide bandwidth sensing of nuclear magnetic Larmor frequency spins resonance.

NV can achieve very high magnetic field sensitivity down to single electron spin sensitivity [4] and the current best magnetic field sensitivity is of 0.9 pT Hz^−1/2^ [5]. Some of these magnetometer techniques have direct sensing applications within biological samples [6]. A nanoMRI method using a single NV center has been successfully applied to achieve the 2D imaging of ^1^H NMR signal with a spatial resolution of 12 nm [7]. These results can be achieved by combining conventional confocal or wide field optical microscopy in conjunction with specifically adapted Nuclear Magnetic Resonance (NMR) methods such as Hanh-echo sequence and universal dynamical decoupling [5], [7].

Optical conventional imaging microscopic techniques based on confocal microscopy and wide field microscopy are however intrinsically diffraction limited (resolution ∼half optical excitation wavelength). To achieve nanometric resolution in the localization of NV with purely optical methods, super-resolution methods based on Stimulated Depletion Emission Microscopy (STED) [8] and Stochastic Optical Reconstruction Microscopy (STORM) [9], [10] must be employed. Therefore alternative methods combining STED and STORM with spin resonance techniques (spin echo sequences) have also been implemented. However, still some hurdles exist for the full deployment of nanoMRI technology [11].

This manuscript provides a summary of the latest works [1], [2], [3] showing nanoscale resolution in localizing NV in diamond with potential applications in magnetic resonance imaging of nuclear and electron spins. One of these methods, FMI, uses the Fourier (or k-space) phase-encoding of the NV electronic spins in a diamond sensor and it has been applied to magnetic field sensing. We will describe the implementation to neural field detection and discusses the potential extensions of these imaging techniques to quantum spin defects in other, possibly more practical material such as silicon carbide (SiC).

We also report on two main methods to achieve this. Fourier Magnetic Imaging (FMI) [1] of NV in diamond and optical super-resolution microscopy methods combined with Nuclear Magnetic Resonance (NMR) methods [2], [3]. FMI acquisition and processing method applied to NV in diamond achieves imaging in the k-vector space providing a 3.5 nm resolution. STED and STORM imaging methods are achieving nanometric localization directly in real space, based on deterministic and stochastic localization of fluorophores.

These methods combined with NMR techniques have also been applied to NV and potentially could provide nanoMRI capabilities and magnetic field sensitivity. NV localization with resolution of 2.4 nm and 27 nm have been demonstrated, respectively. These last two methods have not yet been fully applied to magnetic sensing to assess their potential ultimate sensitivity to resolve other nearby spins. They are however expected to be relevant for direct spin-spin interaction studies.

This manuscript delivers an introduction to this rapidly advancing area and illustrates the case of advanced methods for the plausible and potentially significant applications to neural pathways. This contribution highlights the present advantages and relative performance of these methods. The non-invasive mapping of functional activity in neuronal networks is one possible application of these techniques and we will discuss its current status.

The contribution also discusses the further improvements of the technique including the use of atomic defects in a more probe fabrication friendly material such as SiC and concludes that the subject area is now sufficiently mature to engineering a probe and developing a protocol for practical medical applications especially in neuroimaging.

## Methods

### Fourier magnetic imaging

Arai et al. [1] have developed a fast and accurate scanning probe microscope that operates at room temperature and provides a unique combination of high magnetic field sensitivity and nanometric resolution that coupled to Fourier magnetic resonance imaging (MRI) methods may open new horizons. In conventional MRI, in addition to a static high magnetic field defining the quantization axis, and a radiofrequency pulse exciting the nuclear spins, a magnetic field gradient based on three independently controlled gradient coils is used to link the local spins precession frequency to the spins 3D location **r** and the gradient vector of the magnetic field along the quantization axis. The k-space is defined as the time integral of the time variable magnetic gradient vector, so that the phase acquired over time and space by the spins at a specific location **r** can be written as ϕ(**r**,τ) = 2π**k**(τ)·**r**. The MRI signal is made of a part associated to the initial radiofrequency pulse inducing magnetization of the spins, which constitutes the real image, and a part linked to the spins phase rotation ϕ(**r**, τ), that depends on space and time. Therefore, the real space image is the Inverse Fourier Transform (IFT) of the phase rotation in the k-space. The MRI signals ϕ(**r**,τ) in the k-space values are sampled during the image measurement with a carefully designed time sequence of radiofrequency and gradient pulses. Data from digitized image signals are stored during data acquisition and then mathematically processed to produce the final image. The IFT is applied after k-space acquisition to derive the final image. This approach has been modified from conventional MRI, where the probe is a coil sensing nuclear spins in the sample, while in nanoscale MRI the NV electron spin is a probe.

Thus a key feature of the NV-diamond Fourier imaging method is that the phase encoding is applied to NV spins in the sensor, rather than nuclear spins in the sample as in conventional macro-scale MRI. Phase-encoding of the sensor spins broadens the applicability to many other types of magnetic samples, beyond those where the magnetic fields are created by distributions of weakly-interacting spins, such as current-carrying and ferromagnetic samples, action potentials in neuronal networks and magnetite in many cellular structures.

Pulsed magnetic field gradients [12] are specifically used to phase-encode the spatial information on NV electronic spins in the “k-space” [1]. The wave number space measurement is then followed by a fast Fourier transform to generate nanoscale resolution, wide field-of-view (FoV), compressed sensing speed-up, real-space images. The key advantages versus real-space imaging, are the spatially multiplexed detection [13] enhancing the signal-to-noise ratio (SNR) for characteristic NV centre densities, a high data acquisition rate that compressed sensing can expand [14] and the concurrent acquisition of signal from all the NV centres in the FoV. The FMI pulse series simply consists of a laser initialization pulse, a microwave dynamical-decoupling succession for spin-state manipulation [15], [16] and finally a laser readout pulse.

**Fig. 1:**
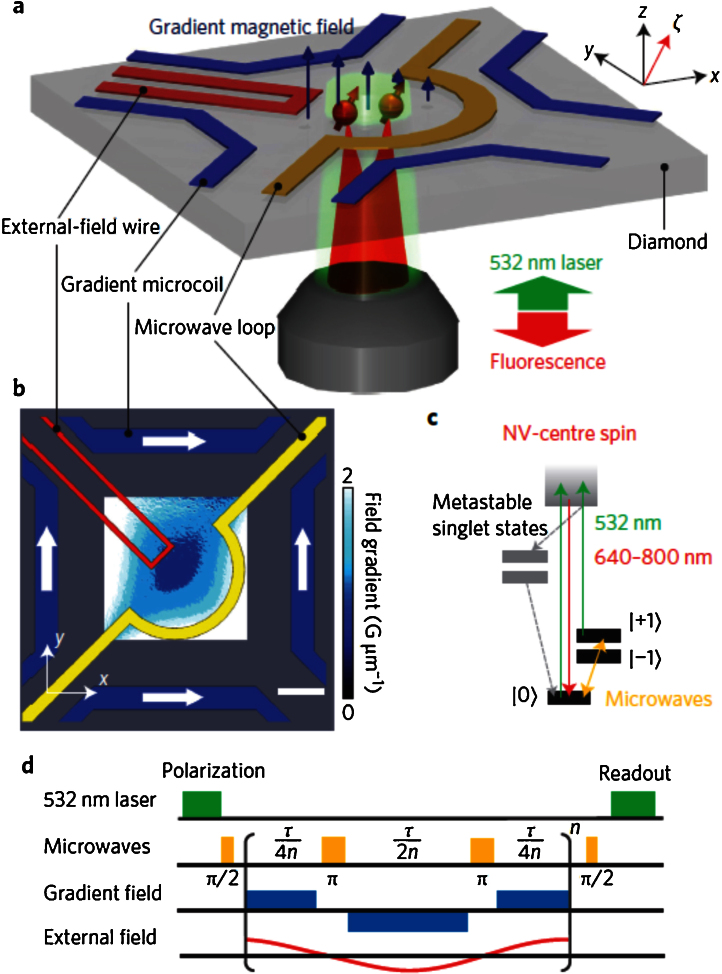
The Nano Fourier Magnetic Imaging Microscope according to Ref. [1]. (a) and (b) are the side view and top view outline of the probe, (c) the NV centre energy level diagram and (d) the imaging sequence. The NV centre magnetic sensors are located near the surface of a diamond chip. A green laser is used to initialize and read out the NV spin states that are coherently manipulated with resonant pulses by using a microwave loop (yellow). The controlled magnetic field gradients for the NV spin phase encoding in k-space are generated by sending currents through pairs of wires (blue). For the principle demonstration an external field wire is used to create a non-uniform DC or AC magnetic field. The NV quantization axis is offset from the surface normal of the diamond sample axis and aligned with a static, uniform magnetic field ≈ 30 G created by a permanent magnet that is not shown in the figure. (For interpretation of the references to color in this figure legend, the reader is referred to the web version of this article.)

Fig. 1 presents the outline of the probe and the imaging sequence; a and b are the side view and top view outline of the probe, c the NV centre energy level diagram and d the imaging sequence. In addition to the usual optical polarization of NV using a pulsed 532 nm laser, the excitation microwave frequency resonant with NV ground state electron spin transition, and the optical read-out of the spin state, a magnetic field gradient is used. The pulsed magnetic field gradient encodes the phase rotation of NV spin before and after a microwave π-pulse in a k-space, as defined in conventional MRI. In addition to test the NV probe as magnetometer, an AC magnetic field is sent and imaged using the NV sensors. The microwave frequency, magnetic field gradient for encoding NV-phase during the π-pulse sequence, and the variable magnetic field to image, are sent via microwave loops, gradient micro coils and external field wire, respectively, directly patterned by e-beam lithography on a polycrystalline diamond coverslip. The sensor itself is a single-crystal diamond grown by chemical vapour deposition and implanted with N ions to form NV centres at a depth estimated between 20 and 100 nm. One sample contained a very low density of NV, and the FMI method was used to image a single NV with 3.5 nm for 1D imaging and 30 nm for 2D imaging. A second sample was fabricated with arrays of nanopillars each containing two NV centres on average. The two NVs were imaged by FMI and were found separated by 121 nm with 9 nm resolution. Acquisition time was less than 20 ms.

This nanopillar containing two NVs precisely located is then used to image the AC magnetic field sent to the external wire with obtaining a magnetic gradient sensitivity of 14 nT/nm/Hz^1/2^. To extend the FoV to 15 × 15 μm^2^ but maintaining the same nanoscale resolution (FoV/resolution = 500), 167 nanopillars were used to image an additional AC field. The imaging was done in a hybrid real and k-space imaging modality across the array of many nanopillars providing a high resolution k-space imaging of the magnetic field patterns, produced by the external wire. The localization of NV centre in several nanopillars was achieved with 30 nm resolution. By employing compressed sensing techniques [15], based on random sampling at a rate lower than the Nyquist rate, a measurement speed-up factor of 16 has been demonstrated without substantial loss of accuracy. An integration of k-space imaging with a wide-field microscope using a complementary metal oxide semiconductor (CMOS) or charge coupled device (CCD) camera can provide rapid imaging across a large FoV with nanoscale resolution.

### Stimulated depletion emission microscopy—optical detected magnetic resonance

STED microscopy was previously shown to be successful to image single NV centre with 6–8 nm resolution [8] by using, in a scanning probe modality, in addition to an excitation beam a second beam (STED-beam) to induce stimulated radiative emission depletion of NV. As this beam has a doughnut beam shape (dark at the centre) superimposed to the Gaussian excitation beam, the NV photo-luminescence is deactivated in part of the diffraction limited spot, thus providing a resolution less than the diffraction limit. An excitation (100 ps pulse width and 8 MHz repetition rate, 0.5 mW) pulsed laser at 532 nm is used, spatially superimposed with a pulsed STED laser (3 ns pulse width and 8 MHz repetition rate, 5W) [2]. The resolution scales with the square root of the power of the STED beam. However the final limit to the resolution cannot be achieved by merely increasing the power. In fact, even if NV is very photo-stable under high power, however the high refractive index of diamond and its planar surface introduce aberration and scattered light that prevent to have a perfect zero field of the STED-beam. To address this issue and increase resolution a solid hemispherical lens in diamond (solid immersion lens, SIL) has been fabricated on the diamond containing NV. The SILs size ranges within 5–8 μm and are directly fabricated within the diamond by focussed ion beams. Two type of diamond were used: the smaller SILs where fabricated in high-purity polycrystalline diamond grown by chemical vapour deposition, while larger SILs were sculpted in a high purity single crystal diamond, where NV centres were generated at a depth of 4 μm by 6 MeV N ions implantation. Optical detected magnetic resonance (ODMR), Rabi and Hanh-echo sequence have been implemented [2], [17] simultaneously with super-resolution of NV, proving the ability to manipulate single NV spins with this resolution and indicating the opportunity to apply this method in conjunction with more complex sequences used in nanoMRI methods. To achieve STED-ODMR the microwave excitation was integrated with an adapted sequence: the NV centre is initialized to a ground state spin state by exposing it to 532 nm light with a few mT DC magnetic field defining the quantisation axis, then a microwave pulse of varying frequency is applied. The spin signal is read out with high spatial resolution by simultaneously illuminating the sample with excitation and STED light. To increase the signal-to-noise ratio, the sequence is repeated typically 104 times for each microwave frequency. It is expected a magnetic field sensitivity similar to what achieved in diffracted limited imaging methods [11], depending on the microwave sequence used and the additional presence of an AC magnetic field or a gradient field. In this case the main impediment could be the excessive distance of NV from other nuclear spins given the size of the SIL and the lack of scalability of SIL fabrication method. This could be overcome by using a free-standing SIL rather than a diamond integrated SIL, thus allowing NV to be in very proximity to a nuclear spins containing samples. The most likely application of this nanoscale imaging methods is in study of spin-spin interaction in quantum computing architectures. The higher photon collection is also improving of a 2.2 factor the acquisition time required to access to the spin information, while the nanoscale optical localization is a real time process.

### Stochastic optical reconstruction Microscopy—Optical detected magnetic resonance

STORM enables fast and super-resolved imaging/localization of single emitters in a wide-field modality, provided that their photoluminescence can be temporally switched “on” and “off”. These methods has allowed to achieve few ten nanometres 3D spatial resolution in cellular imaging [9], [10]. Super-resolving single NV centres with a sub 20 nanometre resolution in a wide-field localization microscope based on the photoluminescence blinking of high-pressure high-temperature nanodiamonds was also demonstrated [18]. In [3] a STORM technique has been developed for NV centres in bulk diamond permitting to perform at the same time NV sub diffraction imaging and ODMR measurements on super-resolved NV spins. Commercial diamond samples of type IIa, grown by chemical vapour deposition were used. The samples contain as grown (depth of up to ≈2 μm from the surface) and artificially created NV by N ion implantation (an average depth of ≈10 nm from the diamond surface).

By applying the STORM method the localization of NV was with 27 nm resolution, limited by the sample drift. An estimated resolution without sample drift is 14 nm. The combination of STORM and spin detection permits to assign spin resonance spectra to individual NVs located at nanometric distances and in a parallel acquisition mode as opposite to probe scanning modality. Therefore spin-STORM methods could allow the implementation of parallel NV sensors for nanometric magnetic resonance imaging in nanoMRI applications.

To perform STORM, the switching “on” and “off” of NV is achieved by adjusting the excitation intensity and wavelength (a CW 594 nm laser with an intensity of 1 kW/cm^2^, far below NV saturation), as at this wavelength NV is brought to switch between a bright and dark state, this last attributed to a different charge state of the defect which does not possess optical spin read out and whose fluorescence is outside the here collected spectral window [3]. This was possible as in bulk diamond NV possesses a homogenous photo-physics dynamics. This approach is particularly interesting as, due to the photo-physics properties of NV, it permits to control the “on” and “off” time of the emitters (4s on time was used for best resolution of 27 nm); longer “on” time in fact permits higher resolution. A high sensitivity CCD camera was used to acquire a sequence of frames with an exposure time set on the average “on” time of the NV. CCD frames are not synchronised with the switching “on” and “off” of the individual NV therefore a post processing procedure of the images allow to super-resolve nm distant NVs. STORM-ODMR was achieved while performing the STORM acquisition imaging sequence by applying to the sample additional microwave pulses, synchronous with the CCD camera frames, and in resonance with the localised NVs electron paramagnetic resonance frequencies. By processing the CCD images it was possible to have 2 NVs super-resolved with the additional spin information. The STORM-ODMR also can be used to enhance the STORM resolution with the square root of the photon number per burst. High-spectral-resolution STORM ODMR has been implemented to reveal the presence of nuclear spins as a test of magnetic field sensitivity of the technique. By applying the microwave frequency with small steps around one specific NV OMDR resonance, the hyperfine interaction of close by nuclear spin (naturally present ^14^N nuclear spins) of a specific nanometric localised NV was revealed. A sensitivity of 190 μT/$\left(\sqrt{\text{Hz}}\right)$ was achieved, 10^3^ time worse than for scanning probe single NV center methods (typically ∼ 180 nT**/**$\sqrt{\text{Hz}}$)[3], due to the low photons counts rate with this low excitation power used in STORM; however STORM-ODMR can have a wide-field of view (100 × 100 μm^2^) and can map with 30 nm resolution the local magnetic field of up to 10^7^ NVs, thus paying off in the terms of eventual total acquisition time in applications. The extension of the methods to nanodiamonds [18] would be important for the field, even if more challenging due to the inhomogeneity of NV photo-physics in nanodiamonds that requires the use of other blinking effects.

### Applications to neural network imaging

NV based magnetometry has been investigated as a non-invasive small magnetic fields measurement of action potentials in neuronal network. In understanding the dynamics of neural network, it is a challenge to resolve the neural dynamics with subcellular spatial resolution or synapse scale resolution. The interest resides in determining the action potential dynamics with single-neuron resolution in whole organisms. Electrophysiology methods are currently used but they are invasive and they cannot achieve both high spatial resolution and wide field of view. A full set of techniques with their figure of merit has been analysed in [19], providing a useful insight of the many techniques available to study neural network activity. The action potential can be translated in a variable small magnetic field that is within NV-based magnetometry, thus providing a non-invasive and non-toxic (no labelling needed) method. The challenge of applying NV-based magnetometry is the typical neuronal pulse duration of 2 ms, peak neural magnetic field value ≤10 nT at 100 nm of axon surface.

In the first study of the application of NV magnetometry to measure the probe sensitivity to axon transmenbrane potential [20], the magnetic field generated by a single axon potential is modelled and this magnetic field has been reproduced by a transmitting current micro wire on the diamond surface, which reproduces the temporal dynamics of an axon. A single crystal ultrapure diamond membrane substrate containing ensemble of NV centres was used. The NV photoluminescence is detected in a wide-field combined with confocal microscope. The magnetic field reading modality was a continuous ODMR or a free induction decay, the two methods providing similar sensitivity of 10 μT Hz^−1/2^. To permit the sensitivity to single axon the method needs to be applied within specific sequence repetition and specific sensing volume (1 μm^3^) depending on the axon size. However even in a not optimised implementation, the NV centres were able to match the spatial structure and temporal dynamics of the simulated neuronal magnetic field.

Using NV-based magnetometry in a very simple setup, the magnetic field produced by an actual potential of a single excised neuron from a marine worm and squid, has been demonstrated [20]. The same has been achieved also external to the whole body of a live opaque marine worm. Therefore currently NV-magnetometry can achieve single neuron scale, whole organism scale, no labelling, 10 nm spatial resolution, 30 μs temporal resolution, 1 mm field of view. The method is non-invasive and non-toxic, allows observation for extended period without adverse effect on the live animal.

For this demonstration a single crystal diamond chip was used with a uniform 13 μm layer of high density of NV (3 × 10^7^ cm^−3^) on the surface, where the biological sample is placed. NV probes are excited in a custom laser-induced fluorescence system by a 532 nm incident laser at a shallow angle from the diamond surface from the other side the sample is placed to have most of the 532 nm, reflected off from the diamond surface to protect the living sample, while the microwave wire loop was on top of the diamond with no effect on the specimen. The NV photoluminescence is collected using aspheric condenser objectives. The magnetic measurements were performed at the same time of electrophysiology measurements, to compare the measured magnetic with the one expected from the measured action potential, proven to be in agreement. The action potential magnetic field was detected as a time varying of the centre of the NV magnetic resonance frequency with temporal resolution of 32 μs and magnetic field sensitivity of 15pT/$\sqrt{\text{Hz}}$. The magnetic field measurement for excised single neuron was peak to peak ∼4nT. Upon changing species, there was no need of changing apparatus or protocol. The diamond chip is also reusable. In the case of whole body, the single neuron action potential magnetic field was peak to peak amplitude ∼1 nT (close to the limit of the magnetic field sensitivity of the here described demonstration), due to the separation of the action inside the animal and the NV sensor layer of ∼1.2 mm. With this method is also possible to determine the action potential propagation direction and determine the action potential conduction velocity.

The current status of neural network activity measurements with NV-based magnetometry can be further improved using the above described super-resolution methods, in particular FMI and STORM-ODMR due to the low laser power used. However the magnetic field sensitivity needs to be improve compared to current realization. This will permit to achieve magnetic field imaging.

## Discussion and conclusions

In Table 1 we show in comparison the NV localisation techniques of the here analysed methods. In addition, we stress that nanoscale localisation of NV can be combined with magnetic resonance imaging at the nanoscale, or magnetic field imaging with nanoscale resolution. Two modalities can be used: k-space and real space by scanning probe and wide field. The first permits to achieve a high FoV at the expenses of localisation, however it provides faster magnetic field imaging due to parallel sensing without losing magnetic field sensitivity. It can also be integrated in a hybrid k-space and real space wide field modality. Scanning probe using STED-ODMR can reach the highest NV localization, but so far magnetic imaging has not been demonstrated albeit the technique could achieve very high magnetic field sensitivity [7]. This technique however does not allow parallel imaging. STORM-ODMR is very attractive as it also allows parallel imaging and it is very suitable for biological applications.

By comparing the techniques we can conclude that so far, [1] provides currently an excellent compromise to achieve nanometric localization, high sensitivity magnetic sensing, with well reduced acquisition time of the magnetic field imaging compared to point-by-point scanning due to parallel imaging. Fourier k-space imaging allows for high SNR detection with spatial multiplexing and high acquisition rate that can be enhanced by compressed sensing. The ability to probe all the NV centres in FoV at the same time can permit to measure the time-correlated dynamics of phenomena across the sample. The technique also has the major advantage of the relative simplicity of the apparatus needed, with the micro gradient coils used for phase encoding integrated with an optical microscope as shown in Fig. 1, a set-up well suited for a mass deployment of the technology. NV centres FMI may allow many applications in life sciences as nanoscale MRI of individual biomolecules in real time, or the non-invasive mapping of functional activity in neuronal networks with synapse scale resolution ∼10 nm and circuit scale FoV >1 mm. Performances of the techniques

These results are however at the cost of a lower NV spatial localization compared to STED techniques. Therefore depending on the application, based on current results, we envision that STED-ODMR and STORM-ODMR techniques can be used to study spin-spin interaction in array of NV spins in a quantum computer architectures (the first) or spin-spin interaction in biological samples (the second). It is also possible to apply SPIN-STED and SPIN-STORM to magnetic resonance imaging at the nanoscale by increasing the microwave sequence complexity.

Further improvements that can be applied to all techniques include optimization of diamond samples for greater magnetic field sensitivity [21], [22], [5], enhanced optical set up for better collection efficiency and spin-state optical contrast [23], extension of NV coherence time via dynamical-decoupling pulse sequence [24]. Specifically for FMI further improvement can be achieved by using smaller micro-coils for stronger magnetic field gradients, parallel real space image acquisition with a wide field CMOS or CCD camera may enable the study of neuronal activity that span length scales from few nm to many mm.

It is important to mention that other methods based on NV-spin sensors have achieved sub-nanometric resolution in nuclear spin spectroscopy/sensitivity. They are based on the use of isolated electron spins (quantum “reporters”) on the diamond surface [25] or ancillary nuclear spins (^15^N spin of NV) [26] coupled to the main NV electron spin sensor. They allow to achieve single proton magnetic resonance detection with about 1 Å resolution [25] and single protein NMR spectroscopy [26] both at room temperature.

In [25], quantum reporters coupled to a nearby NV center are localized with nanometer uncertainty and their spin is manipulated and read out as well. By measuring the quantum reporters spin via NV spin-read out, it is possible to increase the sensitivity to the detection of individual nuclear spin magnetic fields.

In [26], a sensor made of two quantum bits is used to improve the read-out fidelity of the NV electron spin by manipulating both electron and nuclear spins before resetting optically the NV electron spin and using a modified dynamical decoupling sequence. The diamond surface is specially treated to extend 10-fold the spin coherence time of shallow NV centers with proportionate improvement in resolution. The readout fidelity is improved through quantum logic. NV coherence time is increased by wet oxidized chemistry in combination with annealing on the diamond surface, thus increasing the ability to sense single digit numbers of nuclear spins within a single protein. This permits to probe and perform spectroscopy of the isotopically enriched nuclear species within individual ubiquitin proteins attached to the diamond surface and within the NV volume detection. The method allows high confidence detection of individual proteins.

In the context of here discussed relevant application of studying the dynamics of neural network using NV-based magnetometry, these techniques can be directly applied to achieve magnetic field imaging with nm resolution. To be able to sense individual mammals neurons, expected to generate an action potential magnetic field of ∼1 nT, it is necessary to improve current magnetic sensitivity, by using a diamond samples with higher NV concentration (in the case of ensemble measurements) and to extend NV coherence by implementing other above mentioned microwave sequences.

A straight expansion of the FMI and of the other here described techniques, for its further engineering, would be to consider the use of another solid-state quantum spin system based on defects in SiC [27], [28], [29], [30], [31], [32], [33]. SiC is a CMOS-compatible material having an advanced manufacturability that could make much easier the engineering of the probe. As well the lower cost and larger provision and distribution of material would be permit a quicker expansion of the technology. SiC defects have electronic states with sharp optical and spin transitions that make them increasingly relevant for nanoscale sensing. Furthermore SiC benefits from the mature fabrication techniques of a material that is much more widely used in real world applications than diamond. Six distinct defect types in the 4H polytype of SiC (4H-SiC) with electronic bound states working as quantum bits similar to the NV centres were identified [27]. The spin of the electronic bound state can be optically initialized via an infrared laser, coherently controlled by microwaves, and subsequently optically measured via a photoluminescence intensity measurement. [28] demonstrates the isolation and coherent control of individual electron spins in high purity monocrystalline 4H–SiC exhibiting exceptionally long ensemble Hahn-echo spin coherence times of more than 1 ms [29], [30] report the characterization of photoluminescence and optical spin polarization from single silicon vacancies in SiC addressing single spins at room temperature also showing coherent control of a single defect spin with long spin coherence times under ambient conditions. This particular defect could be employed for nanoscale MRI, that could benefit also from its electrical excitation as obtained in other single defects in SiC [31], [32], allowing the engineering of more compact probes. Further DC SiC magnetometry has been proved [33], to be feasible without recurring to the radiofrequency excitation by pure optical excitation with sensitivity of 87 nT$/\sqrt{\text{Hz}}$ and projected sensitivity of 100 fT$/\sqrt{\text{Hz}}$, thus comparable with the NV centre in diamond.

## Figures and Tables

(Reprinted by permission from Macmillan Publishers Ltd: Nature Nanotechnology, 2015, 10:859–864).

**Table 1:** Method comparison.

Methods	Spatial Resolution (nm)	Dimensions	Acquisition time	Magnetic field sensitivity (μT/$\sqrt{Hz}$)	Modality
FMI [1]	3.6	1D	20 ms	∼1.2	k-space, large FoV
FMI [1]	30	2D	20 ms	Not specified	k-space and hybrid k-space and real space wide-field
STED-ODMR [2], [7]	6	1D planar	Real time	unavailable	Real space, Scanning probe
	2.4 nm	1D SIL	Real time	unavailable	
STED [2]	8	2D planar	Real time	unavailable	Real Space, Scanning probe
STORM-ODMR [3]	27 nm	1D	Post-processing of imaging required	190	Real space, Wide-field
NV Nanodiamonds [18] Localtition	20 nm	1D	Post-processing of imaging required	unavailable	Real space, Wide-field
